# Changes in Lower Body Muscular Performance Following a Season of NCAA Division I Men’s Lacrosse

**DOI:** 10.3390/sports7010018

**Published:** 2019-01-09

**Authors:** Scott W. Talpey, Robert Axtell, Elizabeth Gardner, Lachlan James

**Affiliations:** 1 Exercise and Sport Science, School of Health and Life Sciences, Federation University Australia, Ballarat Victoria 3350, Australia; 2 Exercise Science Department, Southern Connecticut State University, New Haven, CT 06515, USA; Axtellr1@southernct.edu; 3 Orthopaedics and Rehabilitation, Yale School of Medicine, Yale University, New Haven, CT 06510, USA; elizabeth.gardner@yale.edu; 4 Department of Rehabilitation, Nutrition, and Sport, School of Allied Health, Latrobe University, Bundoora Victoria 3083, Australia; L.James@latrobe.edu.au

**Keywords:** strength, reactive strength, power, isokinetic

## Abstract

The tactical and technical components of training become a primary emphasis, leaving less time for targeted development of physical qualities that underpin performance during the competition phase of a training program. A deemphasis on physical preparation during the in-season training phase may make athletes more susceptible to injury and decrease performance on the field. Two weeks prior to the start and one week following the conclusion of the 16-week collegiate lacrosse season, lower body force production was assessed in eight National Collegiate Athletic Association (NCAA) Division I Men’s Lacrosse athletes. Lower body force production capabilities were determined via the performance of countermovement jumps (CMJ) and drop jumps (DJ) performed on a force plate and isokinetic strength testing of the quadriceps and hamstring muscle groups across three velocities. Isokinetic strength of the hamstrings and the hamstring to quadriceps strength ratio were maintained or increased over the course of the competition phase of training. Relative peak force obtained from the CMJ and the reactive strength index from the DJ decreased significantly over the season. The maintenance of isokinetic strength and the decrease in CMJ and DJ performance may indicate the presence of neuromuscular fatigue that accumulated over the course of the season.

## 1. Introduction

Balancing athletic, academic, and social stressors during a collegiate competitive schedule presents a difficult programming scenario for coaches. Monitoring changes in the physical qualities that underpin performance over the course of a competitive phase of a training program provides strength and conditioning coaches with valuable information about the effectiveness of their training prescription [1]. Several studies have investigated changes in physical qualities over the course of a competitive phase of training. For example, in a professional sporting context, over the course of a 46-week elite male handball season, fitness measures of upper-body strength and power were reported to significantly increase by 2% and 7%, respectively, while lower body strength, power, and aerobic fitness were maintained [1]. Similar results in professional sport have been reported in rugby league [2], cricket [3], and basketball [4]. However, within the National Collegiate Athletic Association (NCAA) competitive context, athletes are restricted to a maximum of 20 h per week for any athletic-related activity, including strength and conditioning [5]. Adhering to the NCAA mandated hours restrictions presents further programming challenges for strength and conditioning coaches to maintain or enhance physical preparedness during a season. In a study conducted over the course of an 11-week NCAA Division I (DI) Men’s soccer season, significant decreases were reported in lower body strength and lean body mass [6]. Similar findings have also been reported in collegiate wrestling and American football [7,8], providing evidence that maintaining physical fitness over the course of the competition phase of a program presents a difficult challenge for collegiate strength and conditioning coaches.

Lacrosse is an intermittent field-based invasion sport and although movement demands fluctuate substantially between position groups, competition requires athletes to accelerate, decelerate, and execute agility manoeuvres, often while undertaking physical contact [9]. Therefore, a lacrosse athlete’s strength and power capacity are important from a performance and injury prevention context. A common test of lower body power output is the countermovement jump (CMJ). A number of variables can be derived from the CMJ, each indicating a distinct strength quality [10,11]. These variables may be kinetic (i.e., measures of force) or kinematic (measures of velocity), and respond differently depending on the training stress and physiological make-up of the individual. For example, the same strength-power training intervention generated velocity dominate adaptations in stronger subjects, yet more force-based changes in those who are weaker [12]. By exploring a series of variables derived from a CMJ assessment, the practitioner can gain insight into the nature of fatigue on the player. Consequently, specific training interventions can be targeted to develop the muscle function that requires the most attention.

Development of strength and power qualities during the general and specific phases of preparation, and their maintenance during the competitive phase, is a targeted priority for strength and conditioning coaches working within the sport of lacrosse [13]. However, balancing the necessary physiological load to maintain strength and, enhance technical and tactical performance with academic stress presents a challenge. Indeed, the occurrence of injury and illness within NCAA DI athletes has been shown to be two times greater during periods of high academic stress compared to low academic stress [14]. The above findings highlight the need to understand how the athlete’s muscle function changes as a result of fitness or fatigue during phases of high athletic and academic stress typical within the NCAA competitive season. Therefore, the purpose of this investigation was to understand how lower body muscle performance, specifically strength and power, changed over the course of a 16-week collegiate lacrosse season.

## 2. Methods and Materials

### 2.1. Subjects

Eight NCAA DI men’s lacrosse players (age = 19.8 ± 0.99 years; height = 183.9 ± 1.72 cm; body mass 90.54 ± 6.18 kg) participated in this investigation. All participants were free of injury at the time of data collection and did not sustain an injury that required the loss of training or competition during the season. All participants provided informed consent, and the study was approved by both the Southern Connecticut State University and the Yale University research ethics committee. As members of the NCAA DI men’s lacrosse national championship team each participant had an extensive background in the sport. However, their years of experience within the collegiate lacrosse strength and conditioning program varied, as four participants were first year players, two participants were second year players, and two participants were third year players. It should be noted that although all the athletes who participated in this study were active members of the team, only one individual was a starting player. The student-athletes who participated in this investigation competed in the Ivy League, which does not allow “redshirting”, meaning that all participants were eligible to compete during the season.

### 2.2. Procedures

This investigation utilized a prospective, longitudinal, single group study design. Data were collected during the week prior to the start of the competitive schedule and one week following the conclusion of the NCAA DI men’s lacrosse national tournament for a total of 16 weeks between testing sessions. Testing was scheduled during deloading weeks of the athlete’s periodized macrocyle in an attempt to negate the presence of fatigue during data collection. Each testing session began with the athletes completing a standardized warm-up with three minutes of jogging followed by dynamic stretching over a 10 m distance, targeting the musculature of the lower body. Following completion of the dynamic stretching the athletes were provided with two minutes of self-directed preparation to target areas that they perceived had not been adequately addressed during the standardized warm-up. To avoid any potentiating effects of high force actions prior to the performance of the jumps, all jumps were performed prior to the isokinetic strength testing.

#### 2.2.1. Countermovement Jumps

Following completion of the warm-up, athletes completed a set of four submaximal countermovement jumps with the instruction to increase the intensity of each jump so that the final jump was a “near maximal effort”. After two minutes of rest, the athlete performed a set of four maximal CMJs with 10–15 s between attempts on a force plate sampling at 500 Hz (Kistler Quattro Jump, Winterthur, Switzerland). To isolate force production in the lower extremity, each jump was performed with hands placed on hips. The athletes were instructed to perform a countermovement to a self-selected depth and jump for maximal height [15]. The average of the two attempts that produced the greatest jump height as determined by the take-off velocity was calculated and retained for analysis. Specific variables from the CMJ retained for analysis were; jump height (JH), relative peak power (PP), relative peak force (PF), average velocity (Vel), and total impulse.

#### 2.2.2. Drop Jumps

Drop jumps were performed from a 30cm box onto a force plate sampling at 500 Hz (Kistler Quattro Jump, Winterhur, Switzerland). Although the athletes were familiar with the drop jump exercise because it is part of their normal training routine, they were provided with opportunities to practice until sufficient technique was observed by the tester (i.e., not jumping off or stepping down from the box when initiating their jump). For each attempt, the athlete placed their hands on their hips and were provided with the specific instructions to “jump for maximal height and minimal ground contact time” [16]. The athlete performed a minimum of four drop jumps with the attempt that produced the greatest reactive strength index (RSI) score as determined by the jump height from take-off velocity divided by the contact time retained for analysis.

#### 2.2.3. Isokinetic Strength Testing

Isokinetic strength during a concentric muscle action of both the right and left quadriceps and hamstring muscle groups was assessed across three velocities: 60°/s, 180°/s, and 300°/s (Biodex System 3, Biodex Medical Systems, Shirley New York, NY, USA). Athletes were comfortably secured using shin, thigh, pelvic, and upper torso stabilization straps. Athletes were instructed to cross their arms over their chest and not hold the testing chair during repetitions. Prior to testing at each velocity, the athlete completed three submaximal repetitions of increasing effort, followed by five maximal repetitions. One minute of rest was provided prior to increasing the speed. Athletes were verbally encouraged throughout each repetition to ensure a maximal effort was provided. Peak values for both quadriceps and hamstring muscle groups for each limb at each speed were retained for analysis. The reliability of isokinetic strength testing on the Biodex System 3 at the velocities used in this investigation has been previously established [17].

#### 2.2.4. In-Season Strength Training

Athletes completed two structured strength and power training sessions per week, alongside an injury prevention focused neuromuscular training program implemented as part of their pre-practice warm-up throughout the competition phase of training. Strength and power training were combined into the same session and targeted musculature of the lower and upper body for the development of strength and power qualities. During the competition phase of the annual training plan, the intensity and volume of resistance training sessions were manipulated using flexible non-linear periodization. Therefore, each resistance training session was individualized based upon feedback provided from jump-based monitoring, the individual athlete’s training and competition load as well as their daily academic demands. Consequently, higher intensity strength and power training was prescribed when technical/tactical training was performed at a lower intensity, there were fewer competitive and academic demands, and results from jump monitoring indicated the athletes could cope with high intensity training. Typically, these sessions consisted of 3–5 sets of 4–6 repetitions of 85–90% of the athlete’s maximal effort for strength and general power exercises along with moderate to high intensity plyometric exercises. Alternatively, if feedback indicated that the athlete was not in an optimal state to cope with the demands of an intense resistance training session, a lower intensity session was prescribed. In this scenario, a typical session consisted of 2–3 sets of 10–12 repetitions at 60–65% of the athlete’s maximal effort in company with supplementary exercises targeting joint mobility and stability.

### 2.3. Statistical Analysis

All statistics were computed using the Statistical Package for Social Sciences (SPSS) Version 22.0 (IBM, Armonk New York, NY, USA). To determine the test-retest reliability of the measurements obtained from the CMJ and DJ, five collegiate athletes, similar to the participants in the current investigation, performed the same warm-up and jump protocols on two occasions separated by one week using the same equipment as previously described. The intraclass correlation coefficient (ICC) and the coefficient of variation (CV) were calculated to inform about the quality of the data obtained from these two tests.

Prior to the statistical analysis, a Shapiro-Wilk test [18] was conducted to determine the normality of the data to ensure the appropriateness of utilizing parametric statistics. Paired sample t-tests were used to determine if statistically significant differences existed between measures of force production prior to and following the season. Due to the small sample size, Hedges’ g [19] effect sizes were used to describe the magnitude of the difference between preseason and postseason measures.

## 3. Results

All variables from the CMJ and DJ demonstrated excellent test-retest reliability. Specifically, the reliability of the CMJ derived variables were jump height (JH) (ICC = 0.972; CV = 4.2%), relative peak power (PP) (ICC = 0.983; CV = 3.2%), relative peak force (PF) (ICC = 0.972; CV = 2.7), average velocity (ICC = 0.902; CV = 2.4%), and total impulse (ICC = 0.960; CV = 1.5%). Variables derived from the DJ were JH (ICC = 0.991; CV = 2.5%), contact time (CT) (ICC = 0.997; CV = 4.3%), and RSI (ICC = 0.975; CV = 4.3%).

Athlete body mass did not significantly change (*p* = 0.322) over the course of the competitive schedule (preseason = 90.53 ± 6.17 kg; postseason = 89.85 ± 6.5 kg). Table 1. presents changes in the peak torque of the left and right hamstrings and quadriceps across the three speeds of isokinetic strength testing. Additionally, the ratio of hamstring to quadriceps strength at each of the speeds from preseason to postseason testing was calculated and is presented in Table 1. Results from the paired sample t-tests and Hedges-g effect size analysis revealed a statistically significant (*p* = 0.004) large magnitude effect for the change in the hamstring to quadriceps ratio in the left leg when tested at the slowest speed of 60°/s. A statistically non-significant, large magnitude effect was also observed for the change in hamstring to quadriceps ratio on the left leg at the moderate speed of 180° per second. Additionally, a statistically significant (*p* = 0.041) large effect was observed for the change in peak torque of the left hamstring when assessed at 180°/s. When peak torque was assessed at the highest speed of 300°/s, a statistically significant (*p* = 0.038) large effect was observed for the change in peak torque of the right hamstring.

**Figure 1:**
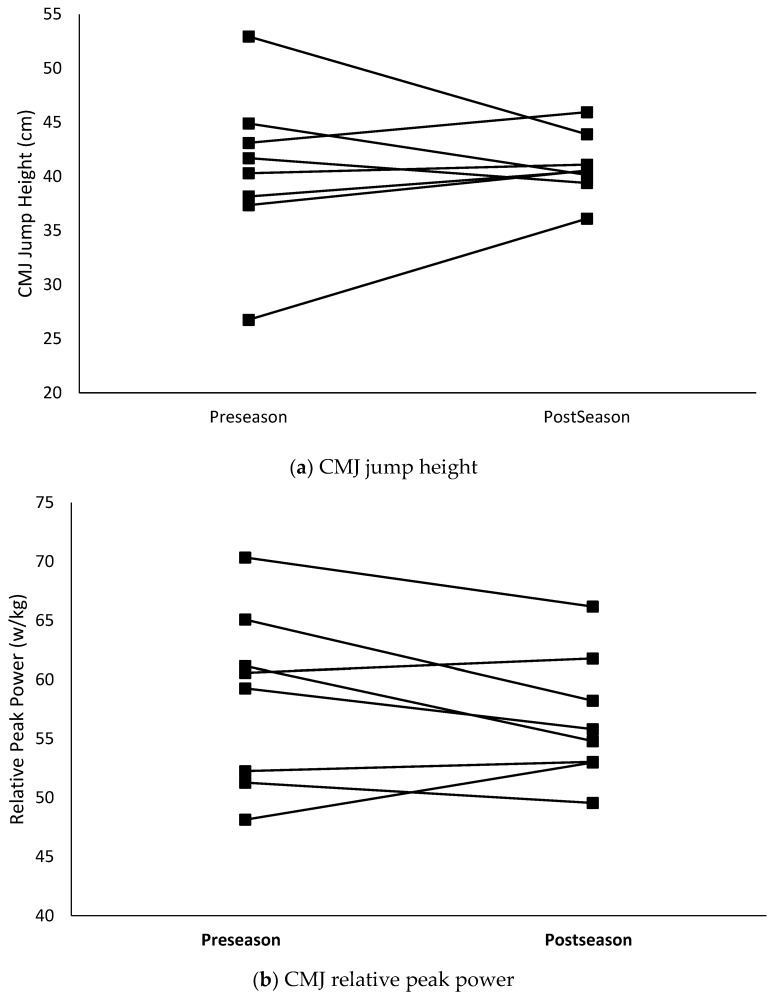

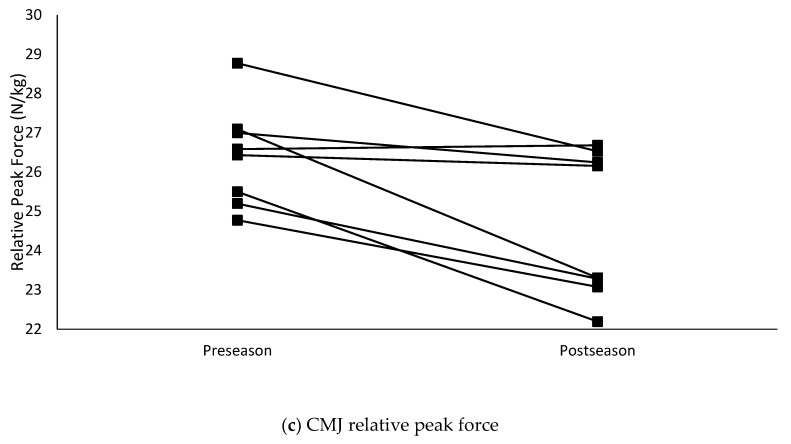
Individual changes in select countermovement jump variables. (**a**) Jump height; (**b**) peak power; (**c**) peak force.

**Figure 2:**
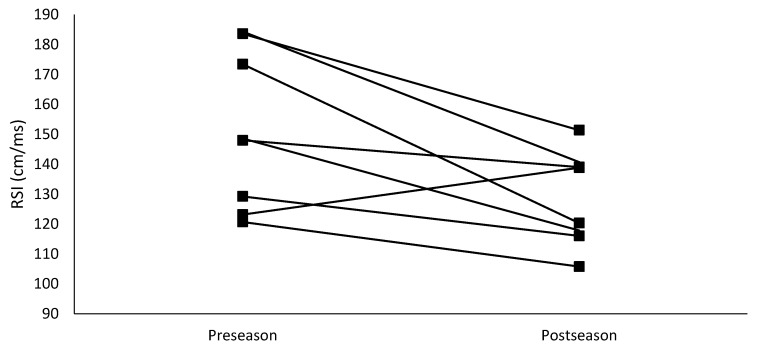
Individual changes in reactive strength index from preseason to postseason.

The relative peak force obtained from the CMJ significantly decreased (*p* < 0.001) over the course of the competitive season. Additionally, although not statistically significant (*p* = 0.07), the contact time during the DJ increased by 14.2%, which was a large effect. The increase in contact time and 1.89% decrease in jump height resulted in a significant (*p* = 0.02) 14.9% decrease in RSI. Group means for the changes in CMJ and DJ variables are presented in Table 2. Individual changes in key CMJ variables of jump height, relative peak power, and relative peak force are graphically displayed in Figure 1 while individual changes in RSI are presented in Figure 2.

## 4. Discussion

This is the first study to assess changes in lower body strength and power qualities over the course of a collegiate lacrosse season. Several of the findings from this investigation will provide valuable knowledge for practitioners currently working with collegiate men’s lacrosse athletes. Isokinetic strength testing utilizes fixed angular velocities to assess force production capabilities at slow, medium, and fast velocities, generally across a single joint. Although isokinetic strength testing is a laboratory based assessment that is not regularly available to strength and conditioning coaches, isokinetic testing provides highly reliable and sensitive information on muscle function that is commonly used to classify injury risk in athlete populations [20,21]. The results from this investigation show increases in the concentric force, producing capabilities of both hamstrings and quadriceps in the right and left legs across slow, medium, and high velocities. The magnitude of the changes in hamstring strength were notably larger than those observed in the quadriceps, particularly at high contraction velocities. The differences in changes between the two muscle groups may be linked to the lacrosse shot technique and the prescription of resistance training specifically targeting the musculature of the hamstrings. Although research into the biomechanics of lacrosse shooting is limited, a previous study has reported that the biceps femoris contributes significantly to force production during the stick acceleration phase when performing a shot at game speed [22]. Game speed shooting was a movement performed by this athlete cohort on a daily basis to improve the technical and tactical components of performance. Another finding of interest is the ratio of the hamstring to quadriceps strength, as these muscle groups work synergistically to dynamically stabilize the knee during athletic tasks, and this measurement is of interest from a lower extremity injury risk perspective [20,23]. A promising finding was that the hamstring to quadriceps ratio improved in both the right and left legs across all three speeds of isokinetic testing, with large and medium effects observed in the left leg at 180°/s and 300°/s respectively. It has been previously reported that a strength ratio of ≥ 60% between hamstrings and quadriceps is recommended for risk reduction of lower extremity injury [20] and that targeted in-season development of hamstring strength can improve this measure in collegiate athletes [23]. The previous findings support the results from the current investigation as strength ratios for this cohort of athletes were within the recommended range and improved with the incorporation of resistance training targeting hamstring strength development.

Assessing the characteristics of different jump patterns can provide practitioners with insight into the force production capabilities of the lower-body that underpin the performance of movements common in sport. Understanding how force production of the lower body is altered over a training phase provides practitioners with valuable feedback about the effectiveness of their program. The CMJ is a commonly used test to assess force production in a slow strength shortening cycle, where movement duration is generally >250 ms, whereas a drop jump is used to assess force production during a fast stretch shortening cycle <250 ms [16,24]. Results from the CMJ indicate that although the athlete’s jump height was maintained over the season, the relative peak force of the group was significantly decreased by 8.6%. Additionally, a non-statistically significant 3.3% decrease in relative peak power output and average velocity were also observed. The finding that jump height was maintained while there was a decrease in force producing capabilities is not surprising, as previous research has demonstrated that an athlete can obtain similar jump heights in a fatigued state compared to a non-fatigued state by altering the mechanics of the jump [25]. The authors hypothesize, based upon the results of the CMJ test, that the athlete’s ability to produce force during a slow stretch shortening cycle was masked by fatigue that accumulated over the course of the season. As CMJ height was maintained while peak force was significantly reduced, these findings indicate that the shape of the generated impulse was altered over the course of the season. Specifically, the shape of the force-time curve likely shifted to a wider, less steep pattern to allow a greater duration of force application. In decisive sporting actions, this wider force-time curve can be considered a disadvantage as the window of opportunity to express force is often limited [26]. These findings are reinforced by those of contact time in the DJ, which displayed a large practical increase over the season. Determination of RSI from a DJ has previously been shown to be a sensitive measure of neuromuscular fatigue [27], highlighting that potentially the explosive force producing capabilities of the lower-body were not detrained, but rather masked by the presence of fatigue accumulated over the course of the season. Interestingly, RSI is a ratio of the jump height and contact time during a drop jump and although not statistically significant, the 14.2% increase in contact time appears to have been the primary driver, leading to a decrease in RSI. Future research may be warranted to investigate the validity of RSI as a tool to monitor neuromuscular fatigue in the sport of lacrosse. This information would be valuable to practitioners as RSI can be determined with a contact mat, which is much more cost effective than a force plate used to determine other measures of fatigue obtained from jump-based testing.

Collectively, these findings indicate that the timing of force expression, rather than the magnitude, was most impacted by the demands of the season. The purpose of ballistic tasks (i.e., the weightlifting derivatives and plyometrics) is to improve timing related measures of muscle function and is well documented to induce minimal fatigue and can therefore be considered as a worthwhile addition to an in-season strength and conditioning program for this population [11,28]. However, the prescription of ballistic tasks should not be at the expense of heavy strength training, as reductions in both high force and high velocity expression have been reported in ballistic only programs [11,29].

The results of this investigation must be considered alongside its limitations. There was only a small sample size of participants in this investigation, therefore, it would be inappropriate to generalize these results to a larger population of collegiate lacrosse players. Future research in this area should be conducted with a larger athlete population that would allow for an in-depth analysis of starters vs non-starters and position groups. Additionally, it should be noted that the standard strength-training program for the involved athletes was one developed with the goals of injury prevention and power maximization in mind. Thus, the results may in fact be muted when compared to less specialized and tailored programs common to other collegiate programs. Although not always practical in an applied research setting, to better understand how physical qualities change over the course of a competition phase of training, testing should include a mid-season test. As well, measurement of perceived academic stress was not recorded during this investigation and thus its effect is unknown. Nevertheless, the results of this investigation may provide impetus for practitioners to include fatigue monitoring within their physical preparation program and for researchers to investigate how athletes cope with the stress associated with rigorous academic and athletic demands.

## 5. Conclusions

Isokinetic strength measures in the quadriceps and hamstrings were either maintained or enhanced over the course of an NCAA Division I men’s lacrosse season. The authors postulate that this was potentially influenced by game speed shooting and running as well as targeted lower extremity resistance training. However, results from the jump based testing potentially indicate the presence of neuromuscular fatigue that accumulated over the course of the competitive season, thus putting them at increased risk for injury and performance decline. This was an expected result as the student-athletes who participated in this investigation were balancing high academic and athletic loads. In addition to carefully tuned strength training, practitioners working with a similar athlete cohort may consider incorporating a fatigue-monitoring program to be proactive and mitigate the risk of the stress associated with being a collegiate student athlete.

## Figures and Tables

**Table 1:** Changes in isokinetic strength measures from preseason to postseason.

	Preseason Mean ± SD	Postseason Mean ± SD	% Change	Effect Size	*p*-Value	95% CI
**60°/s**						
Peak Torque Left Hamstrings (Nm)	134.57 ± 11.8	138.71 ± 17.47	3.07	0.271 (small)	0.052	−9.68, 3.58
Peak Torque Left Quadriceps (Nm)	244.89 ± 34.1	230.19 ± 8.89	−6.00	0.589 (medium)	0.313	−0.14, 21.81
Left Hamstring/Quadriceps ratio (%)	55.45 ± 5.0	60.54 ± 4.69	9.17	1.050 (large)	0.004	−7.97, −2.21
Peak Torque Right Hamstrings (Nm)	141.15 ± 13.28	147.51 ± 19.87	4.51	0.376 (small)	0.287	−14.4, 4.98
Peak Torque Right Quadriceps (Nm)	250.38 ± 49.46	250.58 ± 33.44	0.07	0.004 (small)	0.986	−20.01, 19.71
Right Hamstring/Quadriceps ratio (%)	57.75 ± 9.20	59.21 ± 6.6	2.52	0.182 (small)	0.524	−6.62, 3.69
**180°/s**						
Peak Torque Left Hamstrings (Nm)	96.88 ± 15.82	108.89 ± 13.50	12.39	0.816 (large)	0.041	−17.22, −0.47
Peak Torque Left Quadriceps (Nm)	164.05 ± 24.63	168.98 ± 26.7	3.00	0.191 (small)	0.635	−15.29, 9.99
Left Hamstring/Quadriceps ratio (%)	58.85 ± 6.47	65.10 ± 7.86	11.13	0.868 (large)	0.160	−15.66, 3.16
Peak Torque Right Hamstrings (Nm)	101.55 ± 14.77	113.29 ± 13.69	11.56	0.824 (large)	0.093	−19.09, 1.84
Peak Torque Right Quadriceps (Nm)	170.83 ± 22.30	175.21 ± 18.80	2.56	0.212 (small)	0.473	−13.33, 6.85
Right Hamstring/Quadriceps ratio (%)	60.11 ± 10.88	65.19 ± 9.62	8.45	0.494 (small)	0.238	−14.40, 4.23
**300°/s**						
Peak Torque Left Hamstrings (Nm)	77.91 ± 16.54	88.75 ± 14.91	13.91	0.688 (medium)	0.203	−21.43, 5.46
Peak Torque Left Quadriceps (Nm)	129.68 ± 25.89	136.91 ± 16.40	5.57	0.333 (small)	0.324	−17.71, 7.044
Left Hamstring/Quadriceps ratio (%)	60.31 ± 6.95	64.7 ± 6.24	7.27	0.664 (medium)	0.319	−14.13, 5.31
Peak Torque Right Hamstrings (Nm)	80.60 ± 15.72	96.22 ± 18.57	19.37	0.908 (large)	0.038	−22.1, −0.92
Peak Torque Right Quadriceps (Nm)	123.84 ± 26.30	136.96 ± 13.28	11.49	0.646 (medium)	0.094	−21.59, 2.22
Right Hamstring/Quadriceps ratio (%)	65.9 ± 9.96	70.28 ±11.90	6.64	0.399 (small)	0.295	−14.41, 5.21

**Table 2:** Changes in weight, countermovement jump, and drop jump performance. PP = Peak Power; PF = Peak Force; RSI = Reactive Strength Index.

Test	Variable	Preseason	Postseason	% Change	Effect Size	*p*-Value	95% CI
	Weight (kg)	90.54 (±6.18)	89.86 (±6.57)	−0.75	0.10 (small)	0.32	−0.82, 2.1
**CMJ**	Jump Height (cm)	40.65 (±0.74)	40.94 (±0.29)	0.71	0.48 (small)	0.88	−0.04, 0.04
	Relative PP (W/kg)	58.51 (±5.35)	56.55 (±5.35)	−3.3	0.34 (small)	0.21	−1.41, 5.34
	Relative PF (N/kg)	26.42 (±1.28)	24.29 (±1.88)	−8.6	1.25 (large)	<0.01	0.57, 2.89
	Avg Velocity (m/s)	1.81 (±0.16)	1.74 (±0.12)	−3.86	0.46 (small)	0.185	−0.39, 0.169
	Total Impulse (Ns/kg)	3.60 (±0.28)	3.57 (± 0.15)	−0.83	0.13 (small)	0.695	−0.14, 0.20
**DJ**	Jump Height (cm)	31.17 (± 0.52)	30.58 (±0.58)	−1.89	1.01 (large)	0.82	−0.05, 0.06
	Contact Time (s)	0.21 (±0.03)	0.24 (±0.04)	14.2	0.80 (large)	0.07	−0.06, 0.03
	RSI (cm/s)	151.37(± 26.3)	128.73 (±15.7)	−14.9	0.98 (large)	0.02	4.39, 40.87
